# Brainstem Encephalitis: An Atypical Manifestation of Zika Virus Infection in Brazil

**DOI:** 10.3390/v17060864

**Published:** 2025-06-18

**Authors:** Mateus Santana do Rosário, Pedro Antonio Pereira de Jesus, Italo Andrade Barbosa Lima, Marcos Vinicius Oliveira Francisco, Cleiton Silva Santos, Lorena Cunha Martins, Luiza Vieira Luedy Trindade, Ricardo Khouri, Isadora Cristina de Siqueira

**Affiliations:** 1 Instituto Gonçalo Moniz, Fundação Oswaldo Cruz, Ministério da Saúde, Salvador 40296-710, BA, Brazil; msrosario@uneb.br (M.S.d.R.); ricardo.khouri@fiocruz.br (R.K.); 2 Hospital Geral Roberto Santos, Secretaria Estadual da Saúde da Bahia, Salvador 40301-110, BA, Brazil

**Keywords:** Zika virus, brainstem encephalitis, neurological complications, diagnosis, treatment, case report

## Abstract

Zika virus (ZIKV), once considered a relatively benign pathogen, has emerged as a cause of severe neurological complications, including Guillain-Barrè Syndrome and encephalitis. This report presents the case of a 21-year-old Brazilian woman who initially presented with fever, rash, and arthralgia. Seven days later, she developed confusion, speech impairment, and gait disturbance. Following a tonic-clonic seizure, neurological examination revealed dysphonia, dysarthria and facial palsy, suggestive of brainstem involvement. ZIKV infection was detected by positive IgM serology and a plaque reduction neutralization test. The patient was treated with corticosteroids and antiepileptic drugs, leading to substantial clinical improvement, and discharge after 25 days of hospitalization. This case underscores the neuroinvasive potential of ZIKV and highlights the importance of early recognition and management of atypical neurological manifestations. It also reinforces the need to consider ZIKV in the differential diagnosis of encephalitis, particularly in endemic regions, and contributes to the growing understanding of ZIKV neurotropism and possible therapeutic approaches for severe presentations.

## 1. Introduction

The first documented human case of Zika virus (ZIKV) infection was reported in Nigeria in 1954, followed by sporadic cases in Africa and Asia [1]. The Aedes aegypti mosquito serves as the primary vector for transmitting the virus, contributing to its high transmission potential in regions where the vector is prevalent [2]. In 2015, Brazil experienced a widespread ZIKV outbreak, which led to an unprecedented increase in cases of congenital Zika syndrome and neurological complications [3].

Human ZIKV infection was initially considered a benign and self-limited disease, characterized by low-grade fever, maculopapular rash, myalgia, arthralgia, headache, and conjunctivitis [4]. However, between 2013 and 2017, the World Health Organization reported an uptick in the incidence of Guillain-Barré syndrome (GBS) cases among individuals with confirmed ZIKV infection [5]. Neurological complications following ZIKV infection often present as conditions other than Guillain-Barré syndrome, highlighting the virus’s neuroinvasive potential and a broad spectrum of clinical manifestations, including meningitis, encephalitis, and myelitis [6].

Brainstem encephalitis or rhombencephalitis is a rare condition often characterized by encephalopathy, cranial neuropathies, long tract signs, and cerebellar dysfunction. A variety of causes include infections, parainfective syndromes, and inflammatory disorders such as autoimmune encephalitis and paraneoplastic syndromes [7]. Listeria, herpes, enteroviruses, and Japanese encephalitis virus are the most common infectious agents associated with rombencephalitis. Other arboviruses like dengue, West Nile, and St Louis were also described as potential causes of this uncommon entity [7]. We report an atypical presentation of ZIKV-induced brainstem encephalitis accompanied by extrapyramidal syndrome in a previously healthy young woman.

## 2. Materials and Methods

The patient was initially evaluated in the emergency room of a public general hospital in Salvador, Brazil, and was subsequently admitted to the neurological intensive care unit for specialized management. Biological samples, including blood, urine, and cerebrospinal fluid (CSF), were collected upon patient admission and throughout hospitalization. Comprehensive laboratory investigations were conducted, including hematological, biochemical, and microbiological analyses for bacterial and fungal pathogens.

Molecular diagnostics for arboviral infections (Zika virus, dengue virus, and chikungunya virus) were performed using real-time reverse transcription polymerase chain reaction (RT-PCR) assays on serum, CSF, and urine samples (ZDC multiplex, Bio-Manguinhos).

ZIKV-specific IgM antibodies were detected using an in-house IgM antibody-capture enzyme-linked immunosorbent assay (MAC-ELISA), provided by the Arbovirus Reference Collection (ARC) division of the Centers for Disease Control and Prevention (CDC), following CDC’s established protocol. ZIKV plaque reduction neutralization tests (PRNTs) were also performed using an established protocol [8]. Additional ELISA-based serological assays were conducted to detect anti-chikungunya (CHIKV) and anti-dengue (DENV) IgM antibodies (Euroimmun, Lübeck, Germany). Serological screening for other infectious agents—including human immunodeficiency virus (HIV), hepatitis B and C viruses, cytomegalovirus (CMV), and herpes simplex virus types 1 and 2 (HSV-1/2)—was also performed using standard ELISA protocols.

The patient was also subjected to neuroimaging studies, including cranial computed tomography (CT) and magnetic resonance imaging (MRI) of the brain, to assess the extent of neurological involvement.

## 3. Results

### 3.1. Clinical Presentation

The patient, a 21-year-old non-pregnant Brazilian woman, presented on 28 June 2015 with acute onset of fever, maculopapular rash, pruritus, arthralgia, headache, and edema of the hands and feet. These symptoms resolved within 24 h. However, seven days later, she developed progressive neurological symptoms, including confusion, somnolence, speech impairment, and an inability to walk. A generalized tonic-clonic seizure led to her admission to the emergency ward, where she received intravenous phenytoin.

Following the seizure episode, she was transferred to the neurological intensive care unit due to worsening neurological status. On examination, she exhibited lethargy, right-sided peripheral facial palsy, impaired palatal elevation, dysphonia, dysarthria, severe dysphagia, and marked axial and appendicular rigidity. Despite these findings, muscle strength and deep tendon reflexes were preserved. Due to the severity of dysphagia, a nasoenteral feeding tube was placed.

### 3.2. Laboratory and Imaging Findings

Cerebrospinal fluid (CSF) analysis revealed 2 cells/mm^3^ with a predominance of lymphocytes, protein levels of 43 mg/dL, and glucose levels of 64 mg/dL. Real-time PCR for ZIKV, CHIKV, and DENV was negative in serum, CSF, and urine samples. Specific anti-ZIKV IgM antibodies were detected in serum (MAC-ELISA), and a ZIKV neutralization test (PRNT) was positive with 1:320 titer with a 50% neutralization cutoff. Other serological tests for CHIKV, DENV, HIV, hepatitis B and C, CMV, and herpes simplex virus (HSV-1/2) were negative. Cranial computed tomography (CT) and magnetic resonance imaging (MRI) of the brain revealed no abnormalities.

### 3.3. Follow-Up and Outcome

A diagnosis of brainstem encephalitis with extrapyramidal symptoms was established, and treatment was initiated upon admission with intravenous acyclovir (10 mg/Kg q8h for 10 days) and methylprednisolone (1 g/day for 5 days). The patient showed substantial clinical improvement over the subsequent 34 days. Auditory and visual hallucinations were present during inpatient stay. During the acute phase, the patient was treated with carbamazepine (3 mg/kg/day) and valproate (15 mg/kg/day), both administered via the nasoenteric tube. No further seizures were observed. As her clinical condition gradually improved, antiepileptic medications were progressively tapered during hospitalization, and she was discharged with a planned schedule for complete discontinuation. Modified Rankin Scale (mRs) improved from grade 5 to 2 when she was discharged, and she presented only mild dysarthria at that time. Nine days later, during an outpatient follow-up visit, she was found to be fully clinically recovered. The patient continued to be monitored in an outpatient setting, and nine years after the acute episode, she remains fully functional. Minor residual symptoms persist, including intermittent migraine-like headaches, memory lapses, and mild cognitive impairment.

## 4. Discussion

Rhombencephalitis, also known as brainstem encephalitis, is a rare neurological syndrome with a broad differential diagnosis. The most commonly implicated infectious agents include bacteria (e.g., Listeria, tuberculosis, Borrelia, mycoplasma), viruses (e.g., herpes simplex 1 and 2, enterovirus, Epstein-Barr virus, Japanese encephalitis virus), and less commonly, fungi and parasites [7]. Non-infectious etiologies must also be considered, including post-infectious and autoimmune disorders such as Bickerstaff brainstem encephalitis, myelin oligodendrocyte glycoprotein antibody-associated disease (MOGAD), and other forms of autoimmune encephalitis [7].

The clinical presentation of rhombencephalitis typically includes an altered level of consciousness, cranial nerve neuropathy, pyramidal and cerebellar signs, and, less commonly, epileptic seizures. The interval between the onset of viral infection and the manifestation of neurological symptoms varies according to the underlying cause—generally shorter in infectious etiologies and longer in post-infectious or autoimmune conditions [7].

Although West Nile virus and Japanese encephalitis virus have been reported to cause brainstem encephalitis, it is uncommon for this condition to be associated with arboviral infections [7,9]. While isolated cases of rhombencephalitis have been reported in association with dengue and, more recently, chikungunya virus infections, this condition remains unreported for other arboviruses such as Mayaro virus and others [7,10,11].

We describe a case of rhombencephalitis associated with ZIKV infection that is, to our knowledge, the most thoroughly documented in terms of clinical and laboratory findings, despite the absence of confirmatory neuroimaging. Although a review article has suggested the occurrence of ZIKV-associated rhombencephalitis, the cases illustrated therein are not referenced in the primary literature and lack formal clinical or pathological confirmation [12]. A 2016 case from Brazil described rapid neurological deterioration and death in a patient with encephalitis, but no specific brainstem findings were reported on imaging or clinical examination [13]. Similarly, other documented cases of ZIKV meningoencephalitis—including those in immunocompromised patients—did not demonstrate clear rhombencephalic involvement [12,14]. In the present case, the acute onset of dysphonia, dysphagia, and altered consciousness raised immediate concern for brainstem dysfunction. The neurology team promptly diagnosed rhombencephalitis based on clinical grounds and initiated early high-dose corticosteroid therapy. This timely intervention may have contributed significantly to the patient’s complete functional neurological recovery.

The diagnosis of Zika virus (ZIKV) infection was established through serological testing, based on the detection of ZIKV-specific IgM antibodies and a positive result on the plaque reduction neutralization test (PRNT), the recommended standard for serological confirmation of ZIKV infection. Molecular testing by RT-PCR did not detect viral RNA in any of the analyzed samples. This negative result is likely attributable to the timing of sample collection—12 days after symptom onset—when viremia is typically low or undetectable. Although ZIKV RNA has been shown to persist longer in urine than in serum or cerebrospinal fluid, the RT-PCR assay performed on a urine sample in this case was also negative. This highlights the importance of timing in molecular diagnostics and supports the utility of serological assays, particularly in the subacute phase of infection.

Currently, there is no specific antiviral therapy or approved vaccine available for Zika virus (ZIKV). Management of severe neurological complications is primarily supportive and may include the use of intravenous immunoglobulin and plasmapheresis. Although high-dose corticosteroids are frequently used in clinical practice for ZIKV-associated encephalitis, their efficacy remains unproven [6]. Given the lack of targeted treatment options, emphasis should be placed on the early recognition of neurological complications, regular and thorough neurological assessments, and a multidisciplinary approach to patient care. The involvement of speech therapists, physical therapists, and other rehabilitation specialists is essential. Early neurorehabilitation and neurostimulation strategies may play a critical role in preserving and recovering neurological function [15].

Nine years after the acute infection, the patient reported persistent headaches, cognitive difficulties, and episodes of memory lapses. Although no structural abnormalities were detected on neuroimaging, the persistence of mild cognitive symptoms suggests a possible post-infectious disruption of CNS homeostasis, likely mediated by low-grade neuroinflammation or microglial remodeling, as described in experimental models of arboviral infections [16,17].

## 5. Conclusions

Zika virus (ZIKV) should be recognized as a potential etiological agent of encephalitis, particularly in endemic regions, underscoring the need for heightened clinical awareness and a thorough understanding of its neurological manifestations. Advancements in diagnostic methodologies, timely and targeted clinical management, and strategies to reduce hospitalization duration are critical for mitigating long-term sequelae and reducing healthcare burden. This case report adds to the growing body of evidence highlighting the neuroinvasive potential of ZIKV and reinforces the importance of further research into its capacity to cause severe complications such as brainstem encephalitis. Further studies are needed to elucidate the pathophysiological mechanisms underlying ZIKV-associated neuroinflammation and to guide evidence-based management strategies.

## Data Availability

The data supporting the findings of this case report are not publicly available due to privacy and ethical restrictions. As the study involves a single identifiable patient, data cannot be shared to protect patient confidentiality following institutional and ethical guidelines.
